# Broadband Fourier-Transform Optical Photothermal Infrared Spectroscopy and Imaging

**DOI:** 10.1021/acs.analchem.5c02493

**Published:** 2025-09-11

**Authors:** Aleksandr Razumtcev, Gwendylan A. Turner, Sergey Zayats, Ferenc Borondics, Aris Polyzos, Garth J. Simpson, Hans A. Bechtel

**Affiliations:** † Advanced Light Source Division, 1666 Lawrence Berkeley National Laboratory, Berkeley, California 94720, United States; ‡ Department of Chemistry, 311308 Purdue University, West Lafayette, Indiana 47907, United States; § Molecular Biophysics and Integrated Bioimaging Division, 1666 Lawrence Berkeley National Laboratory, Berkeley, California 94720, United States; ∥ 577002 Photothermal Spectroscopy Corp., Santa Barbara, California 93101, United States; ⊥ 55536 Synchrotron SOLEIL, Saint Aubin 91190, France

## Abstract

Infrared (IR) spectroscopy is a powerful method for mapping chemical heterogeneity on the microscale. Synchrotron IR radiation uniquely provides a high brightness and broad bandwidth to further extend the capabilities of IR spectroscopic imaging. However, the diffraction-limited spatial resolution of IR spectroscopy is insufficient for studies requiring submicrometer spatial differentiation. Optical photothermal IR (O-PTIR) microscopy is a powerful, emerging method that overcomes the IR diffraction limit in IR hyperspectral imaging by employing a modulated IR beam and a visible probe laser beam to detect local temperature-induced modulation at the visible diffraction limit. In this work, we extend the spectral range of photothermal infrared measurements by incorporating a synchrotron IR source, demonstrating a combined synchrotron-based O-PTIR modality that enables high spatial resolution far-field chemical imaging spanning the entire mid-IR range. Both optical- and fluorescence-detected photothermal modalities were performed using a step-scan interferometer, demonstrating improved spectral range (541–4000 cm^–1^) when compared to optical photothermal microscopy with commercial laser sources (800–1800 cm^–1^ for this particular source) and improved spatial resolution, when compared to synchrotron microspectroscopy measurements. Following these initial validation studies, synchrotron Fourier-transform fluorescence-detected photothermal IR spectroscopy in combination with synchrotron microspectroscopy measurements was used to differentiate cells in mouse brain tissue sections, which requires submicron spatial resolutions beyond those accessible by IR spectroscopy alone.

## Graphical abstract

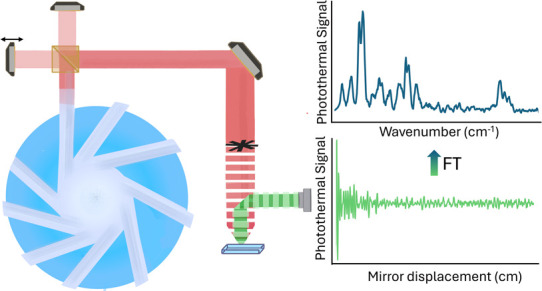

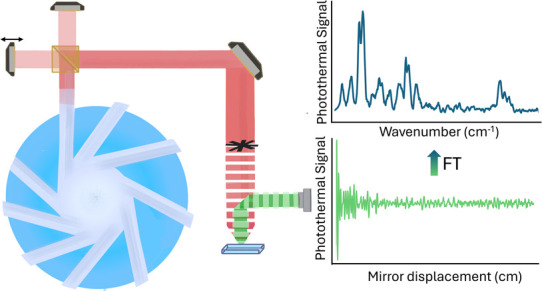

## Introduction

Infrared (IR) spectroscopy is one of the most widely used methods for chemical identification and characterization due to its high specificity and noninvasive nature. IR spectroscopy in the mid-IR region (mid-IR) that spans 4000–650 cm^–1^ (2.5–15 μm) often allows unambiguous discrimination between chemical compounds in a variety of samples, including living cells and organisms, with every major class of biochemical building blocks having distinct spectral signatures (e.g., amide absorption bands for the proteins).^1^ However, the intrinsic diffraction of IR light has limited the spatial resolution of IR-based microspectroscopy to the micrometer regime, hindering measurements of samples with submicron heterogeneity. Such measurements are of potential interest in a variety of fields, ranging from the chemical investigation of subcellular structures to the characterization of nanomaterials.

One common strategy to circumvent the diffraction limit in IR imaging is to perform scanning probe near-field nanospectroscopy. Both scanning near-field optical microscopy (s-SNOM)^2–4^ and photothermal atomic force microscopy (AFM-IR),^5,6^ modalities have been shown to provide tip-limited spatial resolution less than 20 nm. However, while a scanning probe approach has successfully navigated the spatial resolution requirements of some application spaces, many remain that are not sufficiently aided by this technique. Successful measurements are confined to AFM-compatible sample architectures, as sample roughness can cause topographical artifacts and rapid tip degradation. Additionally, comparative studies between near-field IR techniques^7^ and standard spectral libraries have shown imperfect correlation, as the spectral content of AFM-IR techniques can be biased by tip interactions^8^ and possible orientation and polarization effects for near-field IR measurements. Hyperspectral images are usually time-consuming. As a result, applications requiring the investigation of rough samples, large fields of view, or high time resolution are all currently underserved by near-field techniques. There still exists a measurement gap, with a need for a rapid, far-field method to chemically characterize samples with a spatial resolution superior to IR microspectroscopy.

Optically detected photothermal IR (O-PTIR) spectroscopy has made headway in closing this gap over the past decade; the spatial resolution of this novel modality is dictated by the diffraction limit of a visible region probe reporter rather than that of the pump infrared wavelength. The most common mechanism for transducing the photothermal signal is based on temperature-induced variations of the local refractive index, which can be detected in either the transmitted or backscattered direction.^9–13^ With spatial resolutions around 300 nm in commercial systems, O-PTIR has showcased its applicability to a wide range of sample matrices in a variety of fields, including tissue analysis, live-cell imaging, pharmaceutical materials characterization, and microplastics detection, among others.^7,14–20^

Despite the general success and widespread implementation, achievable sensitivities of O-PTIR are intrinsically limited by the relatively weak temperature dependence of refractive index (∼0.01% per °C).^21^ Fluorescence-detected IR photothermal imaging (F-PTIR), which leverages the temperature dependence of fluorescence emission efficiency, has recently arisen as a complementary technique and shown 100-fold improvements in sensitivity when compared to O-PTIR.^22^ By leveraging autofluorescence, F-PTIR can achieve label-free imaging in many samples. Additionally, F-PTIR can also utilize the specificity of fluorescence labeling to capitalize on established labeling architectures to extract chemical information on specific cell types and subcellular organelles.^23^

The mechanism for both the O-PTIR and F-PTIR microscopies is dependent on heat transfer. In short, resonant IR light is absorbed by the material within the IR focal volume. This absorbed energy relaxes and is dissipated as heat. Fluctuations in temperature local to the visible probe volume are then measured via a visible optical property such as refractive index or fluorescence quantum yield. The localization of the IR response with the visible reporter depends on the thermal diffusivity of the sample matrix relative to the time reference frame of the photothermal response, which is often tied back to the IR modulation frequency.^24–27^ In some cases, the colocalization of the visible probe can exceed even the diffraction limit of visible light by probing the temporal dynamics of photothermal relaxation.^28^ For example, using double resonance strategies, colocalization has been pushed even further to single bond localization in fluorescence-detected measurements through the implementation of intramolecular transitions.^29–31^

Despite the improved spatial resolution when compared to diffraction-limited FTIR imaging, the spectral range of optical-based PTIR techniques remains limited due to the availability of commercial IR radiation sources. Because of the low temperature sensitivity of photothermal signal transducers, such as refractive index, a local transient temperature change of several °C is usually required to produce a detectable photothermal response,^21^ and rapid thermal dissipation calls for high repetition rates. Thus, benchtop thermal IR sources do not provide the infrared power necessary to reliably detect changes with modulation. Consequently, the use of high-power, short-pulsed mid-IR laser sources, namely, quantum cascade lasers (QCLs), has become common for photothermal imaging and spectroscopy. Still, the range of a single QCL chip usually covers less than 500 cm^–1^ in the mid-IR region, and not more than 4 chips are installed into commercially available modules. Additionally, although a few research prototypes have achieved lasing in the THz region,^32–35^ currently available commercial QCL sources have a limited spectral range, making it difficult to access frequencies above 3000 cm^–1^ (particularly −NH and −OH stretches) and below 800 cm^–1^.^36^ Furthermore, these QCL sources are an expensive upfront cost, barring widespread utilization outside specialized or otherwise well-funded laboratories.

Synchrotron light sources offer solutions to spectral range limitations and provide an additional avenue of accessibility over commercial laser systems. Every year, synchrotron light sources enable tens of thousands of users across the world to research diverse topics spanning biology, geology, materials science, electrochemistry, and catalysis, among others.^37^ Synchrotron-based IR programs at these light sources provide a spectral brilliance nearly 3 orders of magnitude higher than thermal IR sources used in commercial FTIR spectrometers and a spectral bandwidth spanning the far-IR, mid-IR, near-IR, and beyond.^38^ Synchrotron IR radiation is routinely used for IR microspectroscopy^39–42^ and, more recently, IR nanospectroscopy.^26,43–47^ The spectral brilliance positions the synchrotron IR well for photothermal implementation. Although beamtime each cycle is subject to limitations, these sources are particularly attractive in application spaces where the needed spectral coverage is not available via commercial QCLs, such as in the study of inorganic materials (sub 600 cm^–1^) and in the study of biological materials (over 3000 cm^–1^).

In this work, we demonstrate the combination of synchrotron IR light with optical-based FTIR. The resulting spectroscopy enables subdiffraction spatial resolution and improved sample accommodation and overcomes the bandwidth limitations of laboratory-based IR sources. Moreover, because optically detected PTIR methods do not require detection of infrared photons, this approach avoids the limitations of standard IR detectors, such as mercury cadmium telluride (MCT) detectors, which have low-wavenumber cutoffs, spectrally dependent sensitivities, and nonlinear responses and require cryogenic cooling. Thus, synchrotron IR optically detected PTIR has the potential to enable broadband spectroscopy across the full spectral range, including the THz regime. We achieve broadband photothermal spectroscopy by employing a step-scan Michelson interferometer approach followed by Fourier transformation (FT) of the collected interferogram. Although an FT approach has been used previously to detect the broadband photothermal response with an AFM,^26,47^ the FT approach has not previously been demonstrated with optically detected PTIR. In addition to expanding the spectral range of far-field photothermal measurements, the interferometric approach demonstrated herein has the potential to be adapted to other broadband IR sources. These include other accelerator-based user facilities, benchtop sources with intrinsically high-duty cycle and low peak power, and emerging broadband laser sources with large spectral radiance and repetition rates more amenable to heat dissipation dynamics relevant to the photothermal process.^48,49^ Here, we demonstrate both synchrotron O-PTIR and F-PTIR to enable broadband high-resolution IR imaging of model systems and fluorescently labeled fixed mouse brain tissues.

## Results

Two complementary beam paths were constructed for broadband synchrotron-based PTIR at advanced light source (ALS) beamlines 1.4 and 2.4 to assess the performance of two different signal generation strategies: fluorescence-based F-PTIR and refractive index-based O-PTIR, respectively. Both instruments are described in detail in the Experimental Methods section. In brief, the synchrotron O-PTIR system (Figure 1a) was built around a commercial O-PTIR microscope (mIRage-LS, Photothermal Spectroscopy Corp.) with a flip mirror installed in the beam path to switch between synchrotron and QCL modalities. The F-PTIR system was built around a modified commercial infrared microscope (Nicolet Nic-Plan) equipped with a photomultiplier tube for detection of fluorescence emission in an epi-configuration (Figure 1b).

**Figure 1:**
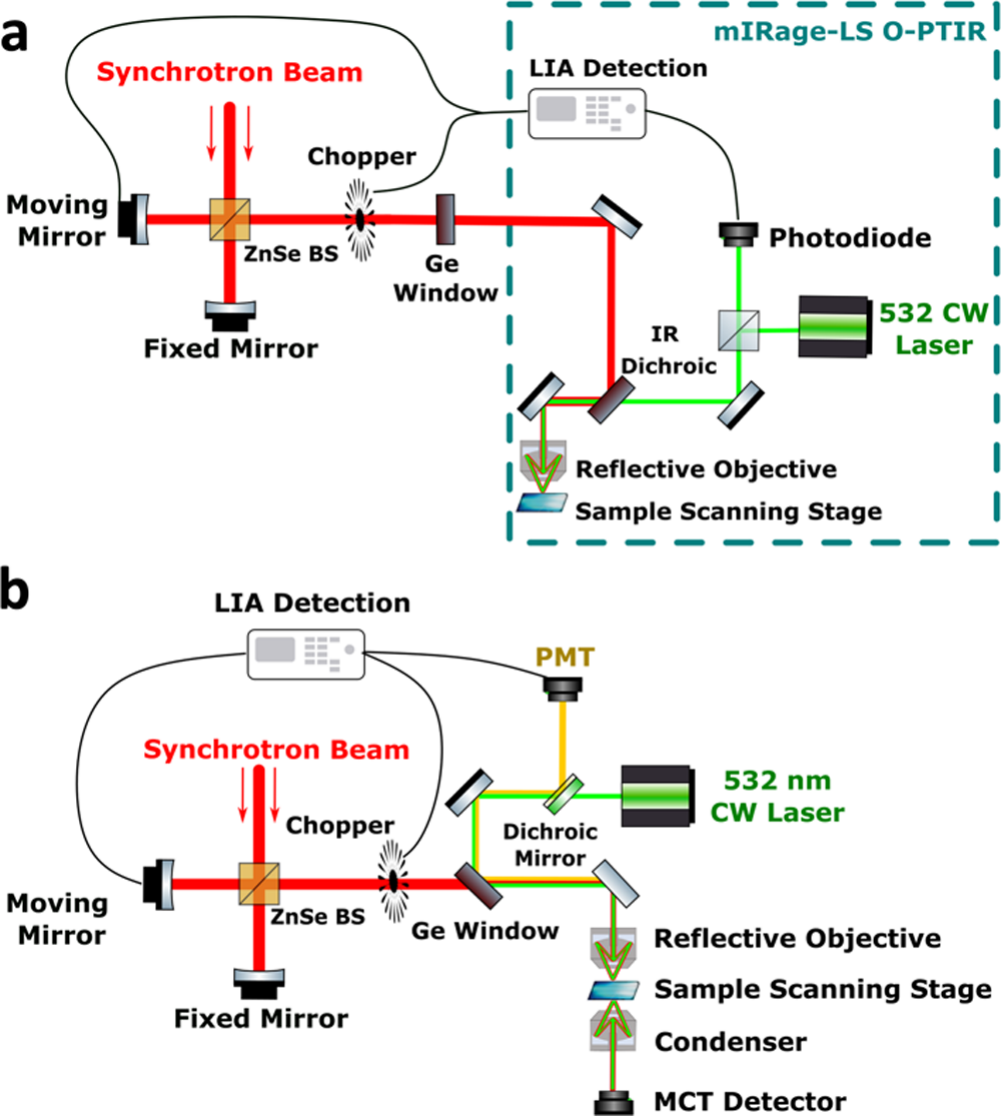
Instrument diagrams for PTIR systems integrated into the ALS beamlines. (a) Instrument used for O-PTIR spectroscopy was built around a commercial O-PTIR system. Broadband synchrotron infrared light passed through an interferometer before entering the microscope. Backscattered 532 nm probe light was demodulated at the chopper frequency at each position of the interferometer to extract the amplitude of the photothermal modulation. (b) Instrument used for F-PTIR spectroscopy and imaging had a similar design, but epi-detected fluorescence emission was used as the photothermal signal reporter. This instrument supported simultaneous transmission FTIR imaging. PMT—photomultiplier tube; LIA—lock-in amplification; BS—beamsplitter.

Prior to entering either microscope, the synchrotron beam was directed to a custom-built step-scan interferometer and an optical chopper. Although ALS synchrotron radiation is inherently pulsed (60 ps), it also has a high repetition rate (500 MHz) relative to commercial QCL sources, which typically operate at between 100 and 300 kHz for O-PTIR measurements. The high repetition rate of the synchrotron light is too fast for sufficient thermal relaxation between pulses, given the thermal diffusivities of the model systems studied, which are on the order of 10^–7^ m^2^ s^–1^.^50–52^ Thus, modulating the light at a slower repetition rate is necessary to successfully observe a thermal gradient. Lock-in detection of the O-PTIR or F-PTIR signal was performed at each position of the interferometer moving mirror in a step-scan approach. A step-scan interferometric approach provided a clear trigger event at each interferometer step for averaging the lock-in detected signal and maintaining compatibility with the optical chopper, which had a maximum speed of 1 kHz. FT of the PTIR signals as a function of the interferometer mirror position produced localized IR absorption spectra over a range of IR frequencies between 500 and 4000 cm^–1^. Although synchrotron-generated radiation at the ALS IR beamlines extends from the visible to the THz regime,^53,54^ the spectral range in the present study is limited by the ZnSe beamsplitter within the interferometer and the Ge window, which was used to cut the visible portion of the synchrotron in the O-PTIR measurement scheme and served as an IR/visible dichroic in combining the beams in the F-PTIR experimental design.

Synchrotron O-PTIR interferograms and resulting broadband IR absorption spectra are shown in Figure 2 for two model polymer materials–poly­(ethylene terephthalate) (PET) in Figure 2a–c and polystyrene (PS) in Figure 2d–f, overlaid with their respective ATR (attenuated total reflection) FTIR and QCL O-PTIR spectra. Fourier-transform broadband O-PTIR (FT-OPTIR) recovered all major fingerprint region absorption features of the studied polymers with the nominal spectral resolution of 8 cm^–1^ corresponding to a 0.0625 cm total distance traveled by the moving mirror. Notably, the C–H stretching vibrations (2800–3150 cm^–1^) and the low-frequency bending vibrations of the aromatic rings for both polymers (536 cm^–1^ for PS and 728 cm^–1^ for PET) that were not accessible with the available QCL spectral range were clearly resolved in synchrotron FTIR and FT-OPTIR. The nominal resolution was selected as a reasonable trade-off between the acquisition time and sufficient spectral fidelity to recover all absorption features of the samples. The reduction in the measured resolution relative to the theoretical value is attributed to a combination of postprocessing and nonideal behavior of the interferometer translational stage. Increased spectral resolution in the FT-OPTIR spectra could be improved by scanning the interferometer mirror for longer distances. The numerical comparison between the spectral resolution of spectra in Figure 2 is provided in Supplemental Table 1.

**Figure 2:**
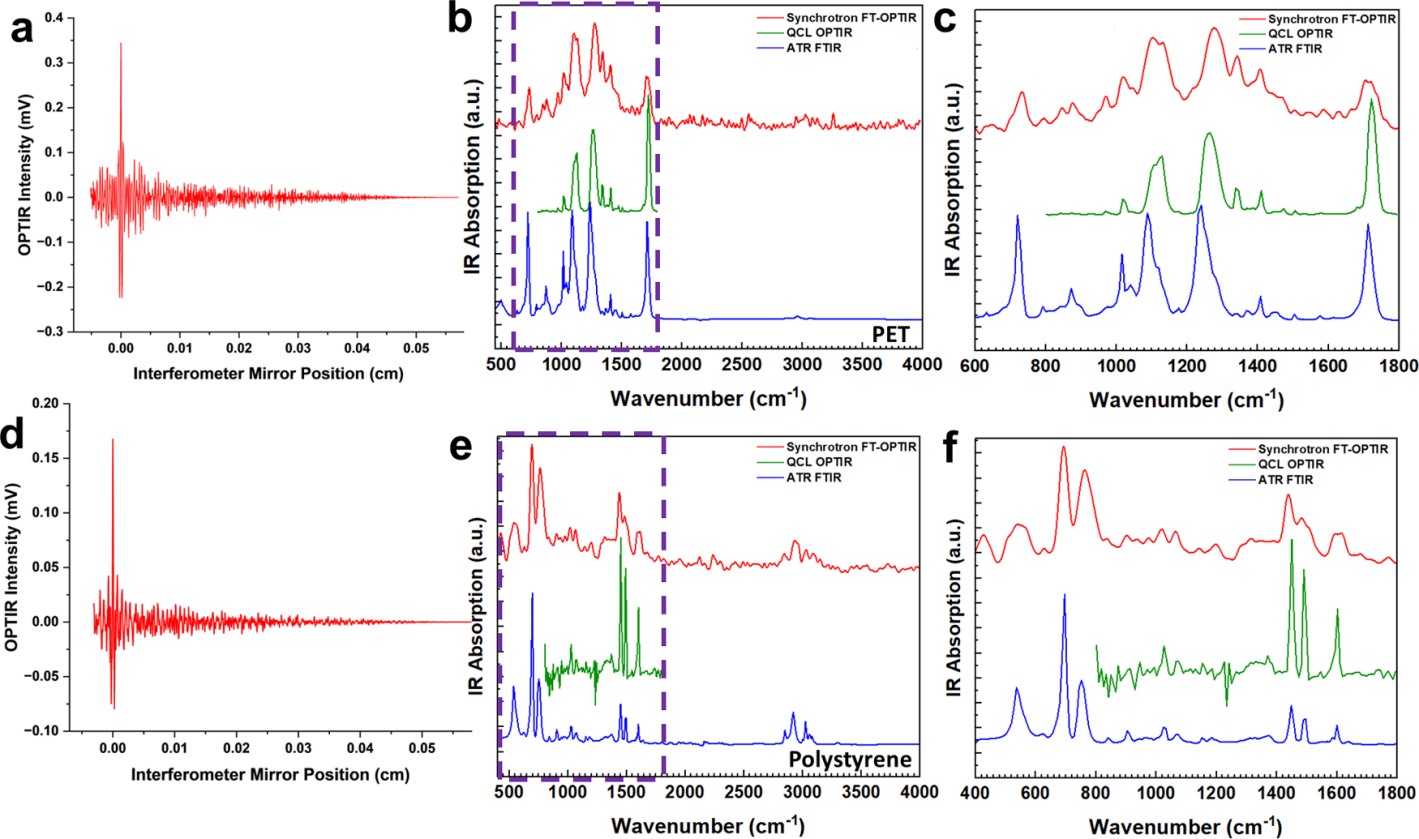
Synchrotron FT-OPTIR single-sided interferograms and the resulting frequency-domain spectra of model polymer materials: PET (a–c) and polystyrene (d–f). An interference pattern in the O-PTIR signal was observed as a function of the interferometer mirror position. The shown interferograms were produced by bandpass filtering and apodization of the raw FT-OPTIR signal. Synchrotron FT-OPTIR significantly extends the range of optical photothermal spectroscopy in both high- and low-frequency parts of the mid-IR spectrum.

The signal-to-noise ratio (SNR) for the most prominent peaks, calculated by dividing the highest intensity of the peak by the average baseline noise within the silent region, is 43 for the polystyrene peak at 1278 cm^–1^ and 52 for the PET peak at 682 cm^–1^. The SNR was calculated for a mean spectrum averaged over 10 individual spectra with 1 s integration time per interferometer position for 1000 interferometer positions. The results of a single spectrum are shown in Figure S3. Several factors likely limited the observed SNR in FT-OPTIR measurements. Physical limitations on the optical chopper used in these experiments capped IR modulation frequencies at <1 kHz, leading to increased flicker noise, also known as 1/*f* measurement noise.^55^ Optical losses, mechanical vibrations, and nonideal beam overlap in the custom interferometer may have also contributed to spectral degradation, particularly in the higher energy portion of the spectrum. Finally, the relatively low IR power of the synchrotron source itself (∼0.5 mW at the sample plane integrated across the whole spectral range) impacted the SNR. Most of these factors have the potential for improvement in future implementations (e.g., by using more advanced interferometers, correcting the IR beam shape with adaptive optics, and planning upgrades to synchrotron infrared beamlines). The observed SNR provides an improvement over hypothetical narrow-band O-PTIR measurements under comparable conditions. The average power of the synchrotron beam was around 0.5 mW at the sample plane after modulation at a 1 kHz frequency. The full wavenumber axis (500–4000 cm^–1^) contained 438 individual wavelength channels with 8 cm^–1^ resolution, resulting in an average power of approximately 1 μW per channel or 2 μW peak power at 50% duty cycle. For comparison, routine measurement parameters in O-PTIR spectroscopy with QCL sources (single wavelength channel, 10 mW average power, 100 ns pulse duration at 100 kHz) yield an approximate peak power of ∼1 W. Hence, assuming a direct proportionality between peak IR power and instantaneous temperature change, the maximum theoretically achievable SNR for QCL O-PTIR/F-PTIR spectra would be roughly 50,000-fold lower if performed at conditions similar to those described in this study. It is important to note, however, that this comparison is oversimplified: like traditional FTIR measurements, the signal of broadband photothermal measurements described herein is multiplexed across all wavelengths reaching the detector, and thus, the SNR for the FT approach is improved by the Fellgett advantage.^56^

In addition to the O-PTIR modality, we demonstrated fluorescence-based F-PTIR detection by modifying an existing IR microscope (Nicolet Nic-Plan) using a Schwarzschild objective with a numerical aperture (NA) of 0.65. The instrument supported simultaneous broadband FT-FPTIR (Figure 3b) and synchrotron FTIR (Figure 3c) imaging by detecting epi-fluorescence emission (Figure 3a) for the former and transmitted IR intensity for the latter. Rhodamine-6G (R6G)-associated silica gel particles were used for initial measurements due to their high IR absorption and easily interpretable mid-IR spectrum. Single-point synchrotron FT-FPTIR spectroscopy maintained an identical spectral range (Figure 3d) with enough spectral fidelity to clearly resolve the absorption peak of silica gel at 1150 cm^–1^. It should be noted that, consistent with previously published results,^22,23^ no vibrational features associated with the fluorophore itself were detected. We note that the synchrotron FTIR spectrum suffers from Mie scattering artifacts, whereas the FT-FPTIR spectrum has fewer scattering artifacts in this case due to the smaller visible probe wavelength.^57^

**Figure 3:**
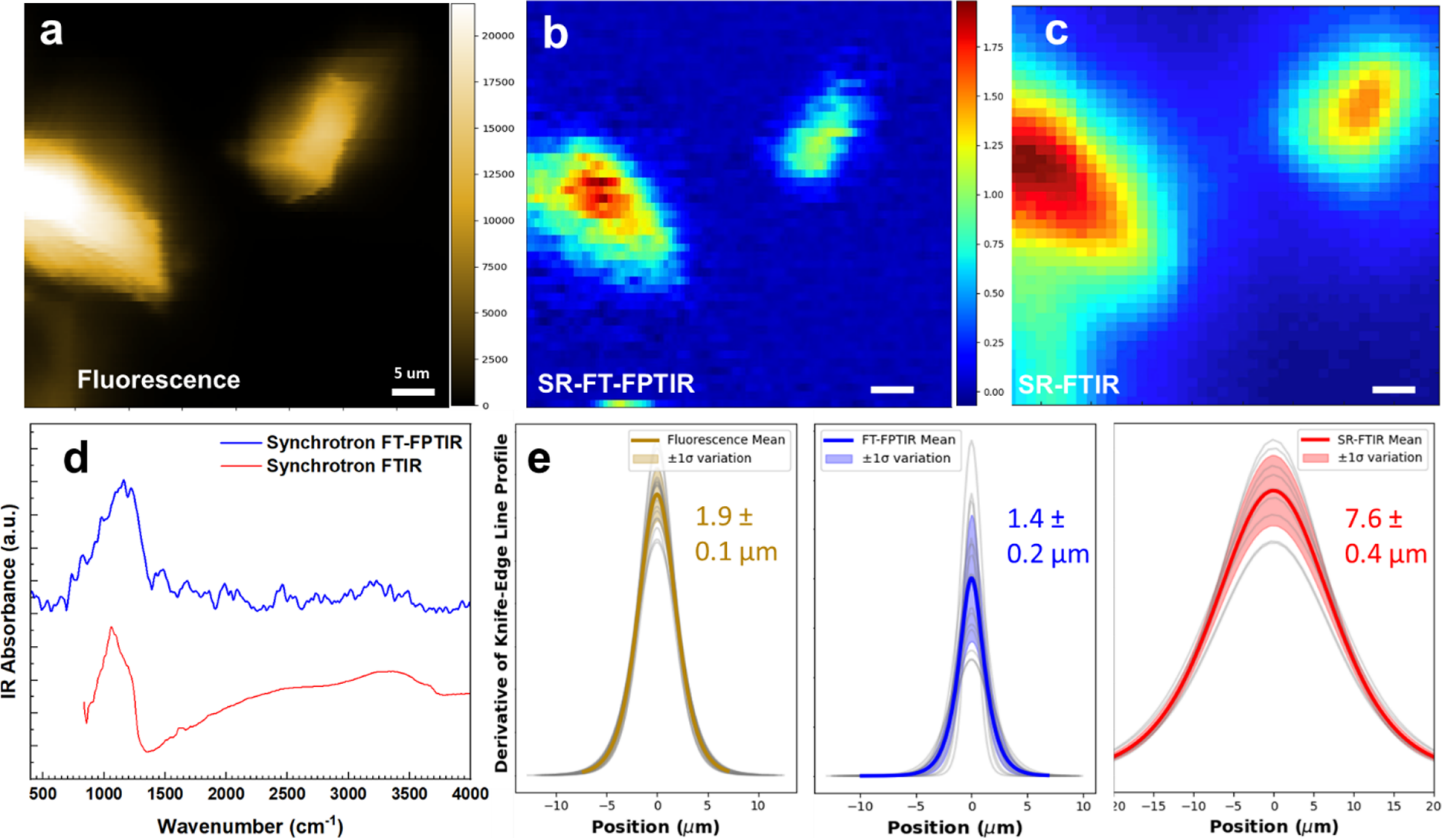
Performance comparison of spectral fidelity and spatial resolution between synchrotron (SR) FT-FPTIR and SR FTIR. (a) Epi-fluorescence image of the studied field of view. (b) FT-FPTIR image and (c) synchrotron FTIR microspectroscopy image slice at 1155 cm^–1^. (d) Representative single-pixel mid-IR absorption spectra for both spectroscopic modalities and (e) calculated resolutions of displayed images demonstrate spatial resolution improvements using a line-profile or “knife-edge” approach on the right particle within the field of view. For each image, the first derivative of the line profile was fit to a Gaussian function, and the fwhm of that function was used to estimate spatial resolution with 95% confidence intervals. Individual fits are shown in gray, with the average and standard deviation in the respective colors.

Following the proof-of-concept synchrotron FT-OPTIR spectroscopy measurements, we evaluated the improvement in the spatial resolution of synchrotron F-PTIR over conventional synchrotron FTIR microscopy. A line-profile, or “knife-edge,” approach was used to calculate the resolution of the right-most particle within the images presented in Figure 3. The Supporting Information features a detailed walk-through of the knife-edge resolution method (Figures S5–S7). A clear spatial resolution improvement is observed in the FT-FPTIR image compared to its FTIR counterpart. For the images below, an average spatial resolution of 1.4 ± 0.2 μm was achieved with synchrotron FT-FPTIR, compared to 7.6 ± 0.4 μm for the diffraction-limited FTIR imaging of the same FoV, resulting in a 5.4-fold resolution improvement at 1150 cm^–1^. We note that the F-PTIR image displays slightly improved estimated spatial resolution when compared to its corresponding fluorescence image. This is likely because the fluorescence image features noticeable astigmatism and out-of-plane fluorescence, which is not as prevalent in the F-PTIR image due to insufficient infrared and visible beam overlap beyond the focal plane. For the 0.65 NA objective, we expect a spatial resolution of ∼6.8 μm fwhm for the diffraction-limited beam at 1150 cm^–1^ under the Abbe diffraction criterion, which is in fair agreement with the FTIR imaging result. Assuming fluorescence emission at 560 nm for R6G, we then could expect a spatial resolution for FT-FPTIR to be as good as ∼430 nm. We note that the knife-edge approach likely underestimates the achieved spatial resolution of the silica gel particles because the particles did not have perfect step edges due to irregularities in the geometry and thickness. Furthermore, the spatial resolution determination in synchrotron FT-FPTIR measurements was pixel count-limited in the vertical direction with a single-pixel size of 1 μm to decrease the acquisition time. Additionally, the laser diode used was likely not a diffraction-limited source. A resolution improvement could be achieved with smaller stage step sizes and in instruments with counterpropagating visible and infrared beams using high NA objectives for both incident beams.

Following the imaging experiments on model fluorescent particles described above, broadband synchrotron FT-FPTIR imaging was performed on fixed thin mouse brain tissue sections labeled by immunofluorescence. The measurements were performed in the striatum region of the brain that has been connected to neuronal death in Huntington’s disease (HD) patients.^58^ Cells were stained by the NucSpot 555 fluorescent dye, which selectively targets cell nuclei, with the resulting fluorescence image shown in Figure 4b. The apparent artifacts in the fluorescence image (Figure 4b) was caused by minor astigmatism in a commercial FTIR microscope that was retrofitted for measurements. The results of broadband photothermal imaging are shown in Figure 4d alongside representative bright-field (Figure 4a), epi-fluorescence (Figure 4b), and diffraction-limited hyperspectral FTIR microscopy (Figure 4c) results of the same FoV. As can be seen in Figure 4, broadband FT-FPTIR enabled selective high-resolution mapping of IR absorption of cell nuclei within the tissue, while the resolution and contrast between cell bodies and surrounding tissue were mostly lost in the conventional FTIR measurements. The FTIR absorption map at 1655 cm^–1^ in Figure 4c corresponds to one of the major fingerprint region signatures of the proteins. FTIR maps at other major mid-IR absorption peaks of biomolecules, including the protein Amide II peak, the nucleic acid phosphate absorption peak, and lipid peaks (Figure S12), show the ubiquitous distribution of major biomolecule classes within the tissue. As such, these spectral bands produce little contrast, which would enable the identification of cell bodies.

**Figure 4:**
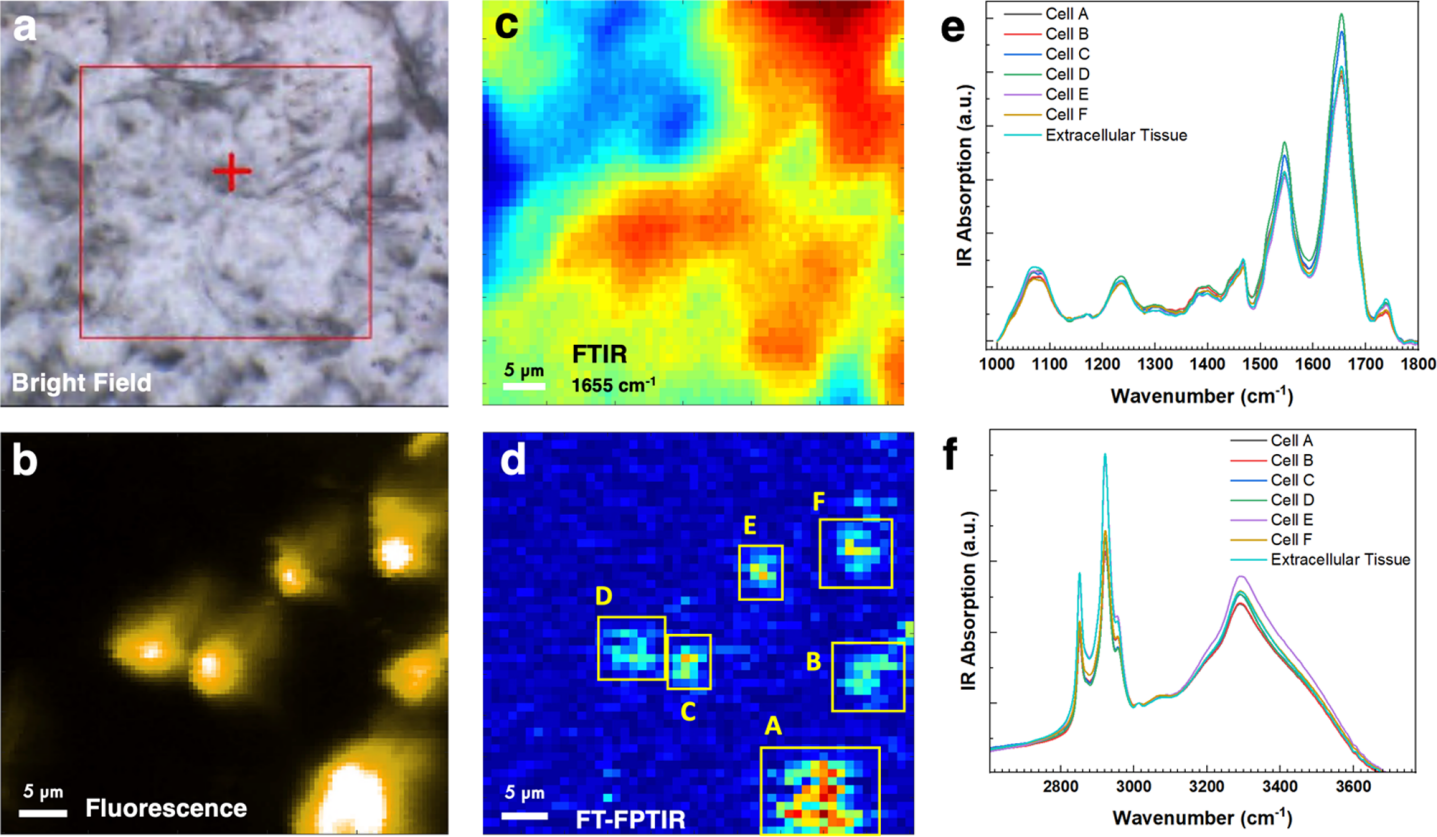
Cell-specific broadband FT-FPTIR chemical imaging of a fluorescently labeled mouse tissue section. The integrated IR absorption FT-FPTIR image shown in (d) only has features coinciding with fluorescence-labeled cell nuclei that can be seen in the fluorescence image shown in (b). At the same time, the contrast between cell bodies and the surrounding tissue is mostly lost in the FTIR results (c) or in the bright-field image (a). FT-FPTIR guided cell-specific synchrotron FTIR spectroscopy results for the extracellular tissue, cell bodies, and a single cell (yellow box in d) for different parts of the mid-IR region are shown in (e) and (f).

Due to the targeted nature, direct connection to IR absorption, and higher spatial resolution of the IR absorption signal, broadband FT-FPTIR absorption maps were used as a guide to create masks for the hyperspectral synchrotron FTIR data set and extract full-range IR absorption information for single-cell bodies. The results shown in Figure 4e,f show variations in IR absorption spectra between the extracellular tissue and the signal arising from individual cells labeled on the FT-FPTIR absorption map in Figure 4d. The variations were relatively minor in the fingerprint region (1000–1800 cm^–1^). However, a larger relative ratio of the lipid absorption peak at 1750 cm^–1^ to the protein amide I spectral signature at 1655 cm^–1^ in the extracellular region (0.16 vs an average of 0.12, Table S3) might indicate a lower relative concentration of lipids within the cell bodies, which is consistent with the striatum being a lipid-rich region of the brain. The most significant spectral variation in targeted FT-FPTIR brain cell analysis was recovered in the high-frequency region (2800–3500 cm^–1^). Spectroscopic analysis in this region was enabled by the improved bandwidth provided by the synchrotron source. As previously mentioned, many of the biochemical infrared absorption signatures in this region are at frequencies inaccessible by commonly available QCL-based narrow-band sources routinely used for photothermal spectroscopy. The ratio of the lipid CH-stretch peak intensities (2850 and 2920 cm^–1^) to the intensity of the protein amide A peak (3300 cm^–1^) (Table S3) was significantly higher for the surrounding tissue (1.76) as compared to the signal originating from any of the individual cells (1.36). The low lipid concentration within brain cell nuclei can be additionally visualized by plotting a ratio map of the lipids to proteins CH-stretch region peaks (Figure S13).

## Discussion

The FT-OPTIR and FT-FPTIR results presented here demonstrate the potential of broadband far-field photothermal spectroscopy. The collected synchrotron FT-OPTIR spectra of model polymer and inorganic materials have features across the spectral range between 500 and 4000 cm^–1^, extending the range substantially over that which is currently accessible with commercial QCL arrays in both higher and lower frequency regimes. This demonstrated spectral range extension afforded by synchrotron radiation has particularly high importance in enabling investigation of new classes of inorganic and biological materials. First, extending the high-frequency cutoff beyond 3000 cm^–1^ is crucial in analyzing the hydroxyl group peak that carries hydrogen bonding and water content information in biological materials, as well as N–H stretches and nonaliphatic C–H stretches. Furthermore, approaching the THz regime, which is also spanned by synchrotron radiation, is crucial for studying phonon vibrations in crystalline materials and inorganic semiconductors, metal–ligand stretching in inorganic complexes, and low-frequency molecular dynamics. It is important to note that the low-frequency cutoff in this work was not dictated by the synchrotron source bandwidth, but rather by the optical transparency of the optical components (ZnSe beamsplitter and Ge window) and could be extended into the far-IR by using different materials with higher transparency.

The potential to perform photothermal IR imaging in the far-IR region of the spectrum is particularly appealing due to the lack of commercially available high-flux laser sources covering this region. While photothermal images at far-IR wavelengths are not presented herein, the achieved spectroscopy indicates this as a future possibility. For a model PS film sample studied in this work, the lowest frequency absorption peak resolved when using a commercial QCL was the vibrational mode at 1452 cm^–1^, while the collected broadband FT-OPTIR spectrum contained resolvable transitions at 755, 686, and 541 cm^–1^. Furthermore, the signal detection in FT-OPTIR spectroscopy relied exclusively on the backscattered visible light, supporting IR microscopy of thick samples with negligible IR transmissivity. Photothermal detection also provides potential improvements in access to the far-IR region over routine FTIR measurements because sensitive far-IR detectors that often require liquid helium are not needed.

In contrast to QCL-based photothermal measurements, single-wavenumber mapping is not supported in this interferometric approach. The interferometer must move the entire length of the interferogram to acquire a spectrum, which is realized by using the Fourier transform of the interferogram. However, the interferometric approach has a distinct advantage over fixed-frequency imaging when time-dependent variations in the signal are of concern. This is particularly applicable to fluorescence-detected photothermal modality. Because interferometric detection is multiplexed by nature, time-dependent photobleaching affects all vibrational modes similarly, preserving the relative peak intensities. Additionally, the influence of photobleaching has the potential to be further reduced with the implementation of standard rapid-scan interferometric detection, which can be accomplished with higher chopping frequencies and standard interferometers. While interferometric multiplexing results in the sample absorbing many wavelengths of broadband synchrotron radiation at once, this did not result in noticeable local overheating and thermal pile-up due to the low peak power of the infrared pump light. This observation is in agreement with previous work that showed that local heating does not exceed 0.5 K for aqueous samples in synchrotron-based IR spectroscopy.^59^

In the present study, the maximum detected signal variation for R6G-associated hydrated silica gel particles was around 0.15%, corresponding to an approximate temperature change of 0.1 K based on the presumed photothermal relationship.^22^ In contrast, performing QCL F-PTIR imaging of the same sample at the absorption peak of silica gel resulted in a temperature increase of 3.0 K by that same relationship. It is important to note that if this interferometric technique is applied using higher power, broadband IR lasing technologies, care and understanding must be taken regarding the delivery of power and thermal loading of the sample.

In addition to the considerable extension of the accessible spectral range discussed above, IR photothermal far-field spectroscopy arguably holds promise for single-cell chemically specific hyperspectral imaging, enabled by the resolution improvement over diffraction-limited mid-IR imaging. Side-by-side comparisons in this work confirm a 5.4-fold spatial resolution improvement for FT-FPTIR with fluorescence detection over FTIR transmission imaging in the same field of view in the fingerprint region at around 9 μm wavelengths. Furthermore, because of the dependence of the photothermal signal on beam overlap and focus, F-PTIR and O-PTIR have improved axial resolution.^27^ In our measurements, this is evidenced by the lack of FT-FPTIR signal from out-of-focus features of the large silica gel particles that are observed in the fluorescence and FTIR images (Figure 3).

One notable advantage of the O-PTIR techniques directly lies in their reliance on visible light for detection, facilitating the analysis of thick or IR-absorbing samples. The limited penetration depth of IR radiation through some materials typically restricts transmission IR measurements, such as water, which, at thicknesses greater than 10 μm, readily absorbs incident IR beams and generally renders measurements in the water absorption region impractical. In contrast, the visible probe beam employed in optical PTIR techniques can traverse thick water layers with negligible attenuation, which removes this constraint. Moreover, instrumentation that is configured for counterpropagation can illuminate samples with IR light from the substrate side, enabling efficient delivery of mid-IR light to surface-adherent biological specimens as long as the samples are thin enough (single-cell layer or tissue sections of corresponding thickness).

In addition to enhancing the measurement sensitivity, F-PTIR also offers targeted IR imaging in complex biological samples, leveraging a vast library of highly specific fluorescent dyes. In this work, brain tissue sections were imaged from mice with HD. HD is a currently untreatable lethal neurodegenerative disease.^60^ One of the known HD mechanisms leading to the loss of cognitive function of the brain is neuronal death in the striatum region.^58^ Investigating the structural and metabolic changes in neurons leading to their death clearly benefits from targeted characterization of this particular cell type in brain tissue, which can be challenging to achieve with commercial FTIR microscopes due to the lack of specificity and insufficient spatial resolution.^61,62^ Herein, as a proof-of-concept demonstration of targeted high-resolution IR imaging of cells in tissue sections, FT-FPTIR analysis was performed on fixed sections labeled with a cell nucleus-specific stain. The FT-FPTIR signal was only generated in the regions labeled by fluorescence and therefore was only observed in pixels coinciding with the locations of cell nuclei in the epi-fluorescence image of the same FoV (Figure 4). No contrast between the cell bodies or nuclei and the surrounding tissues was detected at any major absorption peaks in the FTIR images due to the uniform distribution of major classes of protein and lipid molecules throughout the whole tissue. Using the cell-specific FT-FPTIR result to guide the FTIR analysis enabled extraction of the FTIR signal corresponding to a single-cell body and the ability to spatially differentiate the signal originating from the cell nuclei from the surrounding tissue.

Despite encouraging results obtained in this proof-of-concept demonstration, the FT-PTIR implementation presented herein has room for improvement in future implementations. Relatively low SNR in the collected interferograms and recovered spectra required prolonged averaging at each interferometer position, resulting in long single-spectrum acquisition times (up to 10 min). Such a long single-pixel acquisition time prevented full FoV hyperspectral FT-FPTIR imaging in the current implementation. The contrast in high-resolution FT-FPTIR images was due to the photothermal effect caused by the absorption of the broadband synchrotron light integrated across the entire spectral region, while the spectral information was recovered by conventional low-spatial resolution FTIR imaging of the same FoV. As discussed in the Results section, the SNR in FT-PTIR measurements is expected to be significantly improved in future work by using improved commercial interferometers and synchrotron beam shaping. Additionally, high-speed modulation with fast optical choppers should, in principle, result in significant suppression of the 1/*f* measurement noise, which would also increase the localization of the photothermal signal with the visible probe beam in thermally diffusive systems.^24–27^

In summary, interferometry-based broadband synchrotron FT-PTIR spectroscopy and imaging are demonstrated. FT-PTIR is shown to provide a substantial extension of the accessible IR spectral range in optical photothermal measurements and enable submicron chemical imaging in the fingerprint region. Furthermore, fluorescence-based detection opens additional pathways to perform targeted vibrational spectroscopic imaging in complex biological samples, as demonstrated on mouse brain tissues labeled with a nucleus-specific fluorescence dye. The methodology discussed in this work has the potential to revolutionize synchrotron infrared science by extending far-field synchrotron-based infrared microspectroscopy beyond the diffraction limit. Additionally, these proof-of-concept results suggest that optical photothermal imaging can be realized with broadband sources such as supercontinuum and ultrabroadband IR pulsed lasers. Further technological improvement of such light sources has the potential to open pathways to developing high-speed sensitive chemically specific imaging modalities not requiring access to synchrotron facilities.

## Experimental Methods

### ALS Synchrotron Beam Parameters

All measurements were performed at ALS Beamlines 1.4 and 2.4 (Lawrence Berkeley National Laboratory, Berkeley, CA). During normal user operations, the ALS operates at 1.9 GeV with 500 mA current in top-off mode, producing light spanning from the far-IR to hard X-ray. At the IR beamlines, the X-ray and UV radiation is removed prior to being delivered to the end stations with a series of aluminum- and gold-coated mirrors. A diamond window separates the ultrahigh vacuum of the storage ring from the beamline’s ambient conditions. Approximately 0.5 mW of IR radiation, integrated between 500 and 5000 cm^–1^, was used for the O-PTIR measurements.

### Synchrotron PTIR Spectroscopy and Microscopy Instrumentation

The instrument for broadband FT-OPTIR spectroscopy was a commercial O-PTIR microscope (mIRage-LS manufactured by Photothermal Spectroscopy Corp, Santa Barbara, CA, USA), which was modified in collaboration with the vendor to facilitate beamline integration. Specifically, a flip mirror was installed in the beam path for convenient switching between the synchrotron and QCL (Daylight Solutions) operation modes. Prior to entering the instrument, the IR beam was passed through a custom-built step-scan Michelson interferometer using a ZnSe beamsplitter, where the moving mirror was placed on a linear nanopositioning stage (Aerotech ANT95-L). Except for the interferometer components and a Ge window placed in the beam path to reject the visible component of the synchrotron beam, no additional changes were required in the optics in the mIRage-LS system. A 532 nm CW laser (Hubner) was used as a probe beam, and a Si photodiode was used to detect the scattered light and extract the O-PTIR signal. The mIRage-LS was switched to its normal QCL-based operation mode to collect reference QCL O-PTIR spectra at the same position in which the SR-based measurements were performed.

The microscope used for synchrotron F-PTIR measurements was built around a Nicolet Nic-Plan IR microscope. The side port of the microscope was used to couple in a fluorescence excitation light coming from a 532 nm laser diode (CPS532, Thorlabs). The fluorescence emission was detected in an epi-configuration and filtered from the excitation light by a 550 short-pass dichroic mirror (Thorlabs), followed by a combination of fluorescence filters (532, Edmund Optics; and 550 long-pass filter, Thorlabs). A liquid-nitrogen-cooled MCT/A (Kolmar) detector was used to detect the transmitted IR beam for diffraction-limited FTIR imaging. The synchrotron IR beam and the fluorescence excitation beam were combined on a germanium window (Thorlabs) and focused on the sample with a 32 × 0.65 NA reflective objective (SpectraTech Inc.). The average visible excitation laser power was ∼0.5 mW. The sample was raster-scanned using a Prior Scientific Instruments XY microscope stage H101A to generate 50 × 50 pixel images with 50 × 50 μm fields of view with a single-pixel dwell time of 1 ms for fluorescence imaging and 500 ms for FT-FPTIR imaging.

In both cases, the synchrotron IR beam was chopped by a mechanical chopper (Stanford Research Systems) and placed before the interferometer, at frequencies ranging from 200 to 700 Hz. The PTIR signal at each interferometer mirror position was extracted using a lock-in amplifier (Zurich Instruments MFLI for the O-PTIR system and Stanford Research Systems SR810 for the F-PTIR system). The signal integration time per interferometer position varied between 200 ms and 1 s. The interferometer moving mirror traversed a total distance of 0.0625 cm with 1000 intermediate steps.

### Data Collection and Analysis

For the O-PTIR system, the signal was demodulated at the chopper modulation frequency by using a Zurich Instruments lock-in amplifier at each position of the interferometer to produce experimental O-PTIR interferograms. The interferograms were high-pass filtered to remove low-frequency baseline drift using the OriginLab data analysis software package and then apodized with the Happ–Genzel function. The processed interferograms were then Fourier-transformed, and the amplitude of the transform result was extracted and plotted as an IR absorption intensity. This processing is shown graphically in Figure S2. The symmetry of a single-pass interferogram is evaluated in Figure S4.

In the F-PTIR system, the raw PMT (Hamamatsu) signal was preamplified (Stanford Research SR560) and demodulated at the chopper frequency using an SRS SR810 lock-in amplifier. The signal was then digitized using an AlazarTech ATS9462 digital oscilloscope card. Fluorescence and photothermal imaging employed line scan triggering, resulting in continuous line acquisitions in discrete vertical steps. Resolution measurements for these two modalities employed only horizontal lines to avoid nonuniform pixel sizes. A custom MATLAB script was used to control the microscope stage and reconstruct pixel images for a 50 × 50 μm fields-of-view.

### FTIR Spectroscopy and Hyperspectral Imaging

Reference FTIR spectra for all materials were collected by using a Nicolet iS50 FTIR spectrometer (Thermo Fisher Scientific). The spectra for polystyrene and PET were acquired in the ATR mode using the built-in thermal IR source in the range between 400 and 5000 cm^–1^ with a 4 cm^–1^ spectral resolution.

Synchrotron FTIR microspectroscopy of the silica gel particles and brain tissue sections was conducted at the ALS Beamline 1.4 using a Nicolet iS50 spectrometer (Thermo Fisher Scientific) and a Nicolet Nic-Plan microscope. The IR absorption maps were collected with a 1 μm step for both the *x* and *y* axes, and each spectrum was collected in the 600–5000 cm^–1^ spectral range with 4 cm^–1^ spectral resolution. Acquisition was triggered on a per-pixel basis, and resolution estimates were performed using both the horizontal and vertical directions since the directions were nominally equivalent.

### Sample Preparation

Thin films of PET and PS were provided by Photothermal Spectroscopy Inc.

R6G-associated silica gel particles were prepared by mixing 100 mg of silica gel (60–200 μm particles, SiliCycle) and 5 mg of R6G in 10 mL of deionized water (1.05 mM R6G concentration) and air-drying the extracted particles. Silica gel was ground with a mortar and pestle prior to labeling to reduce the average particle size.

Coronal mouse brain sections were cryo-embedded in Optical Cutting Temperature Compound (OCT) at −80 °C and sliced into 15 μm sections using a Leica Cryostat set at −14 °C. The resulting sections were fixed onto CaF_2_ slides (Crystan, UK. Part #CAFP25-1) with 4% paraformaldehyde for 20 min at room temperature, washed three times in phosphate-buffered saline solution (pH 7.2), and permeabilized. OCT Compound was not observed in the infrared spectrum and likely eliminated during the fixation and rinsing process. Lipofuscin autofluorescence was quenched using Trueblack (Biotium, USA; Part#23007) in 70% ethanol, and NucSpot 555 (Biotum) was applied to the tissue at a 30,000-fold dilution for nuclei staining. Tissues were rinsed with phosphate-buffered saline, refixed with 4% PFA, and kept frozen at −4 °C until FTIR and F-PTIR measurements.

## Supplementary Material



## Notes

Synchrotron infrared spectral range; FT-PTIR spectroscopy measurement processing; FT-FPTIR imaging spatial resolution analysis; and mid-IR spectroscopic imaging of mouse brain tissue sections (PDF)

## ∇

Pixelligent Technologies 6411 Beckley St, Baltimore, MD 21224

## Abbreviations

AFM: atomic force microscopy
FALS: advanced light source
ATR: attenuated total reflection
FTIR: Fourier transform infrared
F-PTIR: fluorescence-detected photothermal infrared
FT-FPTIR: Fourier transform fluorescence-detected infrared
O-PTIR: optically detected photothermal infrared
PTIR: photothermal infrared
QCL: quantum cascade laser
R6g: Rhodamine-6g
