# Supplementary material for: Broadband Fourier-Transform Optical Photothermal Infrared Spectroscopy and Imaging
Source: Anal Chem. 2025 Sep 11;97(37):20117–26. doi: 10.1021/acs.analchem.5c02493 (PMC12461683; doi:10.1021/acs.analchem.5c02493)
Supplement: Supplementary file 1 [file ac5c02493_si_001.pdf]

Supporting Information

Broadband Fourier-Transform Optical Photothermal Infrared Spectroscopy and Imaging

Aleksandr Razumtcev[1,2†], Gwendylan A. Turner[1,2,3], Sergey Zayats[4], Ferenc Borondics[5], Aris Polyzos[3], Garth J. Simpson[2*+], and Hans A. Bechtel[1,*]

[1]Advanced Light Source Division, Lawrence Berkeley National Laboratory, Berkeley, CA 94720
[2]Department of Chemistry, Purdue University, West Lafayette, IN 47907
[3]Molecular Biophysics and Integrated Bioimaging Division, Lawrence Berkeley National Laboratory, Berkeley, CA 94720
[4]Photothermal Spectroscopy Corp., Santa Barbara, CA 93101
[5]Synchrotron SOLEIL, Saint Aubin, France 91190

Table of contents

1. **Synchrotron infrared spectral range**
2. **Additional supporting data for full-range FT-PTIR spectroscopy measurements**
   Processing of FT-PTIR interferograms…………………………………………...S3
   Single-pass FT-PTIR spectrum………..…………………………………………...S4
   FT-PTIR interferogram symmetry…...…………………………………………...S5
   IR Spectral Resolution Comparison…...………………………………………….S6
3. **Additional supporting data for FT-FPTIR imaging**
   Spatial resolution improvement of FT-FPTIR imaging over FTIR microspectroscopy……………………………………………………………......S7
   An example of an FT-FPTIR interferogram…....……………………………...S9
4. **Additional supporting data for mid-IR spectroscopic imaging of mouse brain tissue sections ……..…………………………………………………………......S10**

1. Synchrotron infrared spectral range

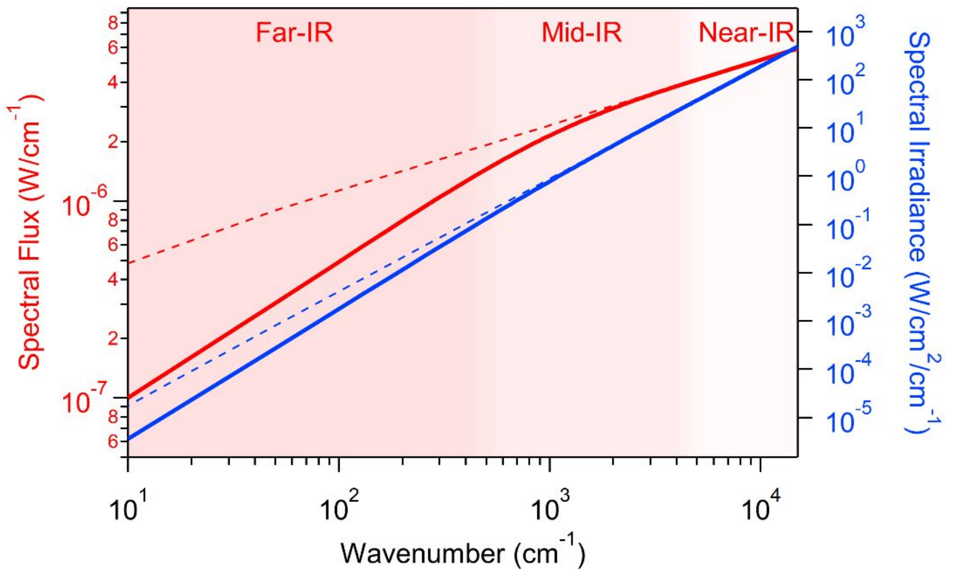

Figure S1. Synchrotron IR radiation spans the far-IR to near-IR and beyond. Calculated spectral flux (red) and spectral irradiance (blue) for bend magnet radiation at the ALS, assuming a collection angle of 17 (V) mrad x 69 (H) mrad (solid line), which describes Beamline 2.4, and 80 (V) mrad x 69 (H) mrad (dotted lines), which enables the maximum extraction of the infrared light to 20 cm^{-1}. The spectral flux was calculated with the SRW [1] package using the accelerator parameters of the ALS and the spectral irradiance (Spectral Flux/Area) was calculated assuming a diffraction-limited spot size (diameter = 1.22λ/NA) for NA = 0.65. The effects of diffraction dominate the decrease of spectral irradiance in the far-IR.

[1] O. Chubar, P. Elleaume, "Accurate and Efficient Computation of Synchrotron Radiation In The Near Field Region", proc. of the EPAC98 Conference, 22-26 June 1998, p.1177-1179

2. Additional supporting data for full-range FT-PTIR spectroscopy measurements

Processing of FT-PTIR interferograms.

Before Fourier transformation to recover frequency-domain IR absorption spectra, raw synchrotron FT-PTIR interferograms were digitally filtered to remove slow baseline drift during the measurements. Following the filtering and DC offset removal, every single-pass interferogram was centered (assigning the centerburst location to zero interferometer mirror position) and apodized using the standard Happ-Genzel function. FT-PTIR interferograms before and after processing are shown in **Fig. S2** below.

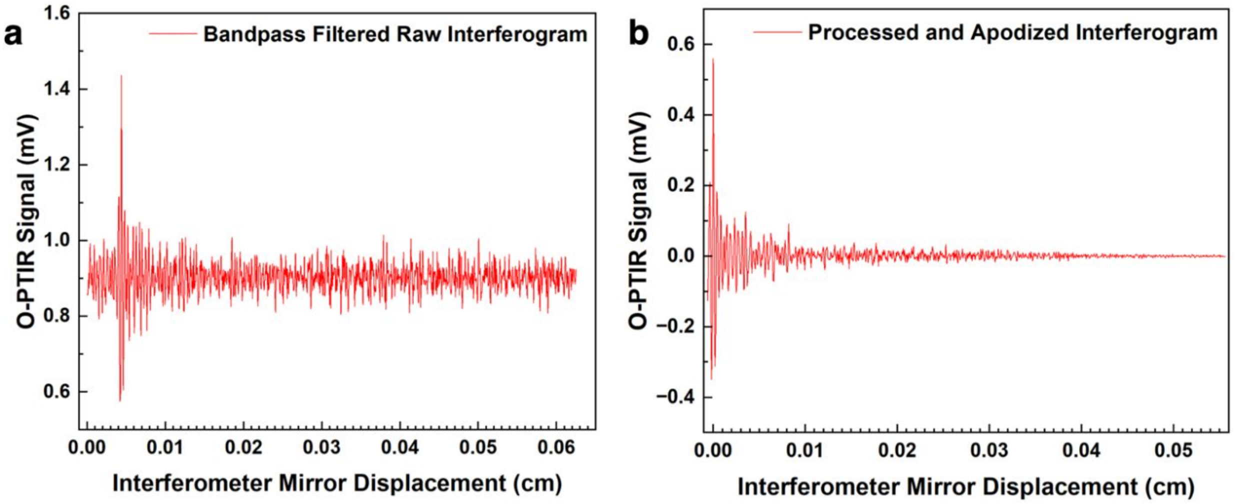

Figure S2. FT-PTIR interferogram processing. (a) – A single raw interferogram before digital processing. (b) – The same interferogram after centering and apodization.

Single pass FT-PTIR spectrum

The spectra of polystyrene, PET, and silica gel shown in the main manuscript are each an average spectrum over 10 individual spectra corresponding to a single one-way pass of the interferometer mirror. An individual FT-PTIR spectrum for PET is given in **Fig. S3** below for reference.

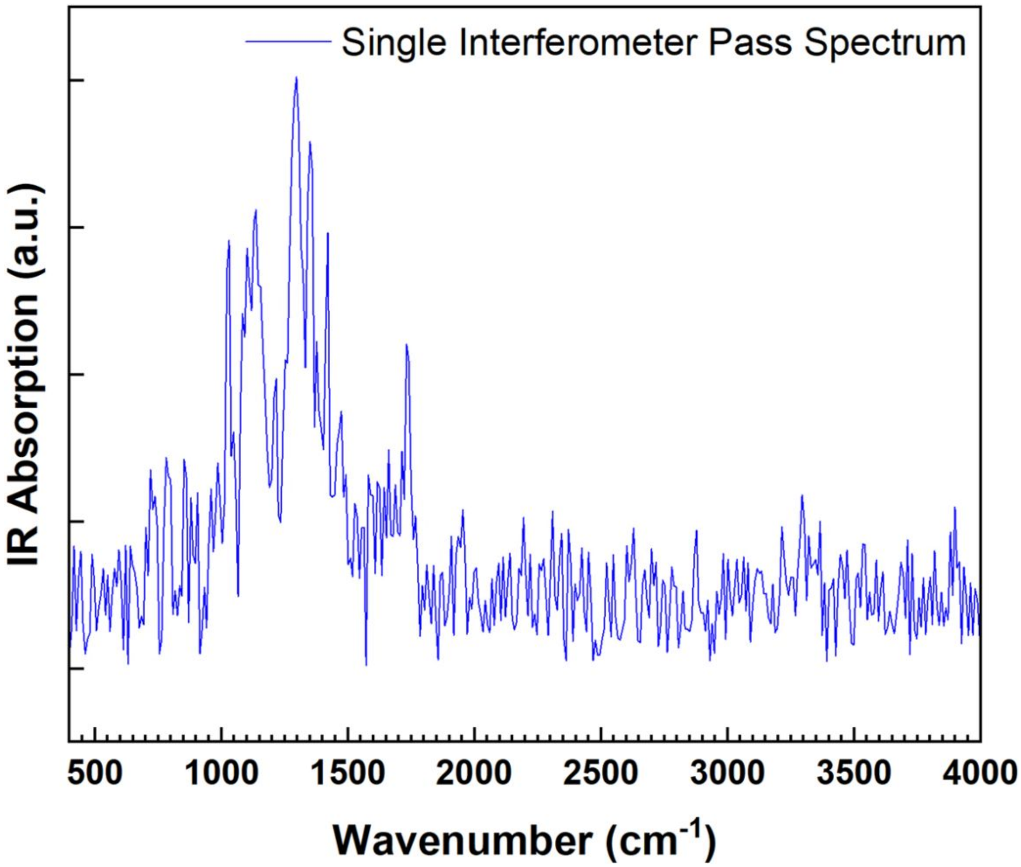

Figure S3. A single unprocessed full-range FT-PTIR spectrum of PET.

FT-PTIR interferogram symmetry

In **Figure S4**, a zoom-in is provided over the centerburst of an FT-PTIR interferogram of PET showing both positive and negative regions of the moving mirror position to illustrate the degree of symmetry of the obtained FT-PTIR interferograms.

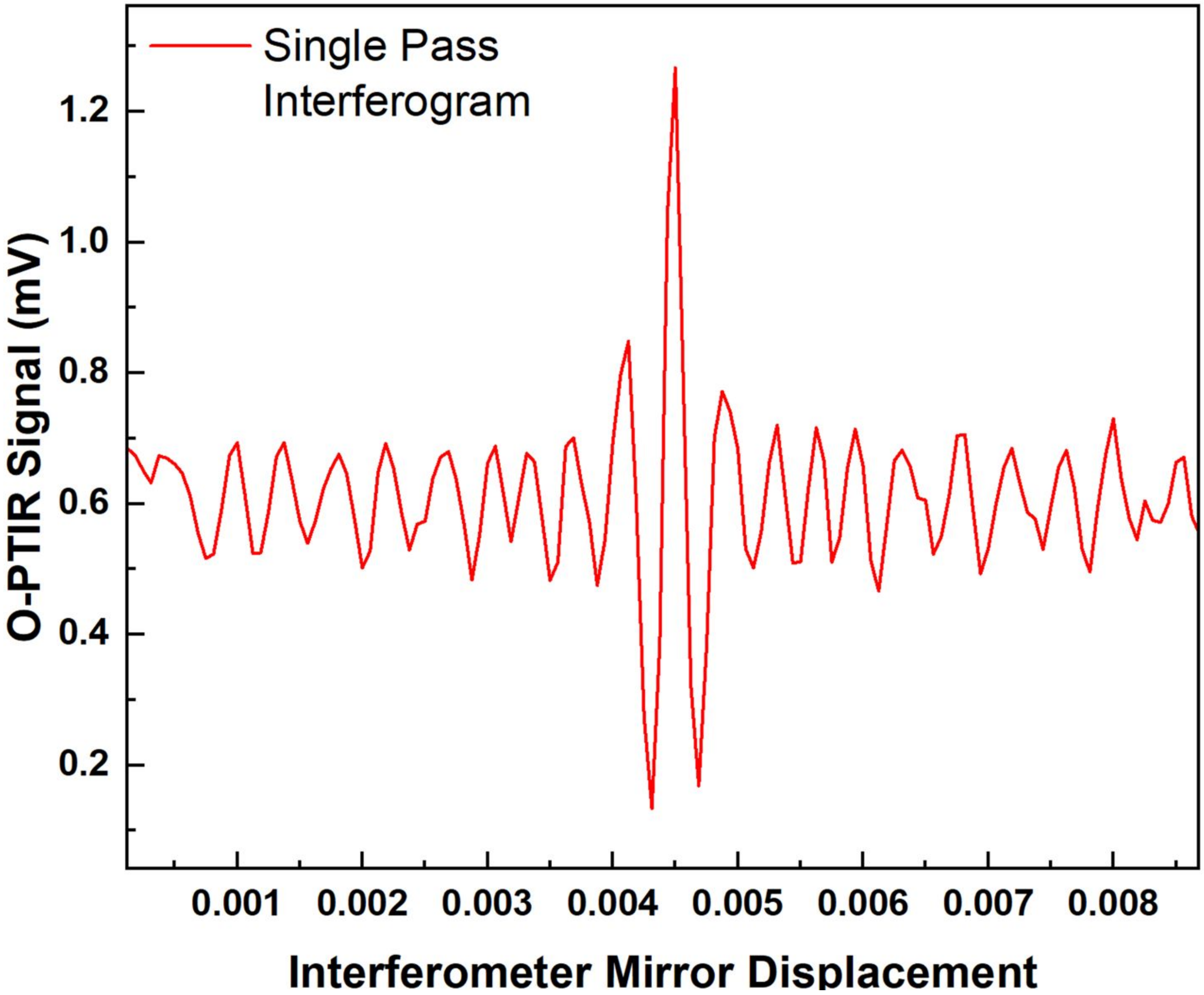

Figure S4. Centerburst region of the FT-PTIR interferogram of PET.

IR Spectra Spectral Resolution Comparison.

Spectral resolution was calculated as a full width at half maximum (FWHM) of a Gaussian function fit to the corresponding peak. Spectral resolution comparison for all main spectral peaks for PET and PS for all three studied spectroscopy modalities is compared in the **Table S1** below:

Table S1. Benchmark comparisons for FT-OPTIR, QCL-OPTIR and FTIR.

Spectral Resolution			
**Spectral Peak, cm^{-1}**	**Peak FWHM, FT-OPTIR (cm^{-1})**	**Peak FWHM, QCL-OPTIR (cm^{-1})**	**Peak FWHM, ATR FTIR (cm^{-1})**
**PET, 1020.**	17	6	10
**PET, 1100.**	45	23	37
**PET, 1270.**	43	40	39
**PET, 1340.**	17	13	Not resolved
**PET, 1410.**	14	11	9
**PS,536**	52	N/A	36
**PS, 680.**	21	N/A	14
**PS, 1450.**	18	12	13
**PS,2859**	41 *	N/A	15
**PS,2919**	55*	N/A	31
**PS,3025**	24*	N/A	14
**PS, 3059**	36*	N/A	12
**PS, 3081**	23*	N/A	12
*Positional error in the home-built interferometer creates spectral artifacts, which is particularly evident in higher frequency components			
**Other Relevant Acquisition Benchmarks**			
	**FT-OPTIR**	**QCL-OPTIR**	**ATR FTIR**
**Achievable Spectral Resolution**	Determined by interferometer arm length. While spectral range is not affected, acquisition time is lengthened.	Limited by QCL step size and QCL bandwidth (typically < 1 cm^{-1}). More QCL steps leads to longer acquisition times.	Determined by interferometer arm length. While spectral range is not affected, acquisition time is lengthened.
**Spectral Range**	THz-near IR. Detection is wavelength-agnostic.	800-1800 cm^{-1} Options exist for silent region or C-H stretch region. However, values above 3000 cm^{-1} are less accessible.	THz-near IR, He-cooled detectors needed for THz regime.
**Spatial Resolution**	Visible-probe diffraction limited	Visible-probe diffraction limited	ATR crystal size, can be IR diffraction limited
**Spectral Acquisition time at 4cm^{-1} resolution**	10 minutes **can be improved with higher IR power*	<1 minute	<1 minute

3. Additional supporting data for FT-FPTIR imaging

Spatial resolution improvement of FT-FPTIR imaging over FTIR microspectroscopy

Spatial resolution for FT-FPTIR and FTIR imaging modalities was determined using a knife-edge method. It should be noted that the knife-edge approach can bias the resolution to lower values (i.e., longer distances) when the sample itself exhibits structure, and therefore should be interpreted as providing a lower bound on the resolution. A field-of-view containing a single small particle (**Fig. S5**) was selected to minimize the effect of an out-of-plane signal. To increase the robustness of analysis, we used a Python script to automatically analyze every edge and its derivative in both vertical and horizontal direction. The results for the FT-FPTIR image analysis with vertical and horizontal edges profiles and their respective derivatives profiles are shown in **Fig. S6** and the results for the FTIR image are shown in **Fig. S7**. All source code for

image processing is archived and available at https://github.itap.purdue.edu/Simpson-Laboratory-for-Nonlinear-Optics/.

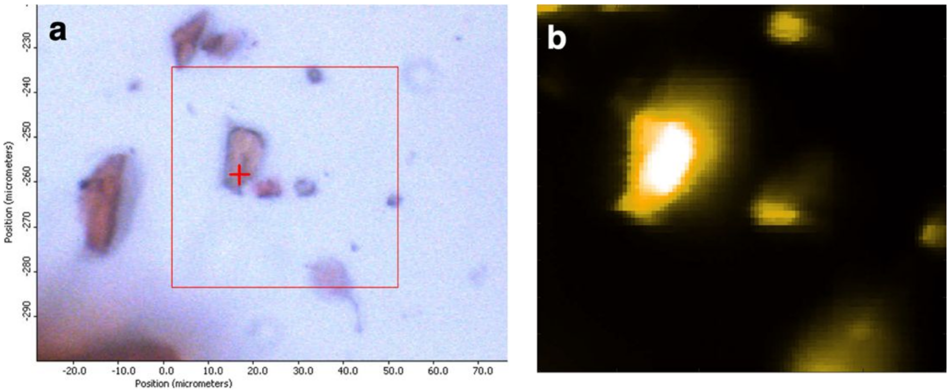

Figure S5. Bright-field (a) and epi-fluorescence (b) images of the FoV used for resolution estimation.

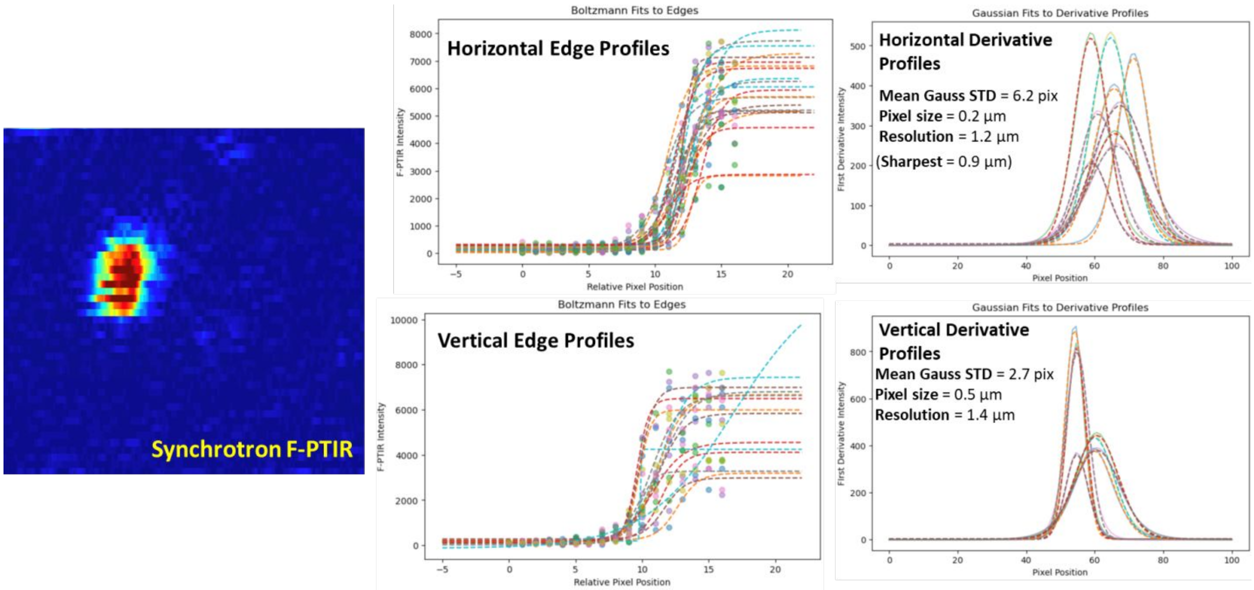

Figure S6. Knife-edge resolution analysis of the FT-PTIR image of a small silica gel particle.

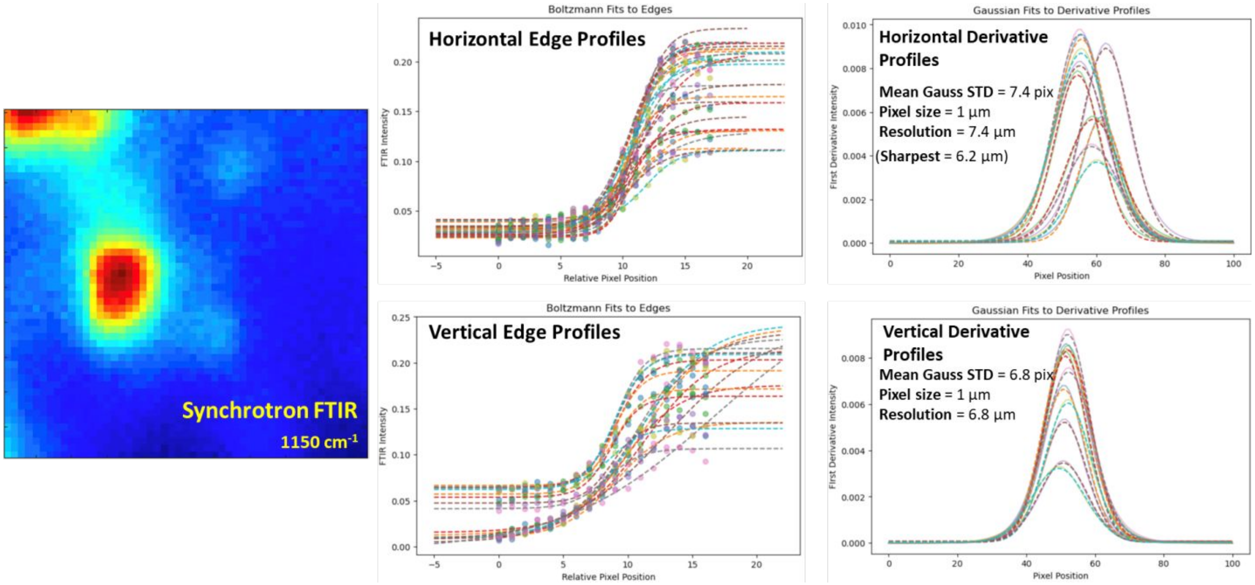

Figure S7. Knife-edge resolution analysis of the FTIR image of a small silica gel particle.

Additionally, FT-FPTIR imaging and profile analysis were conducted for a sample containing dried standard 10µm fluorescent polystyrene beads with the measured bead size of 10.3µm in the FT-FPTIR imaging (**Fig. S8**):

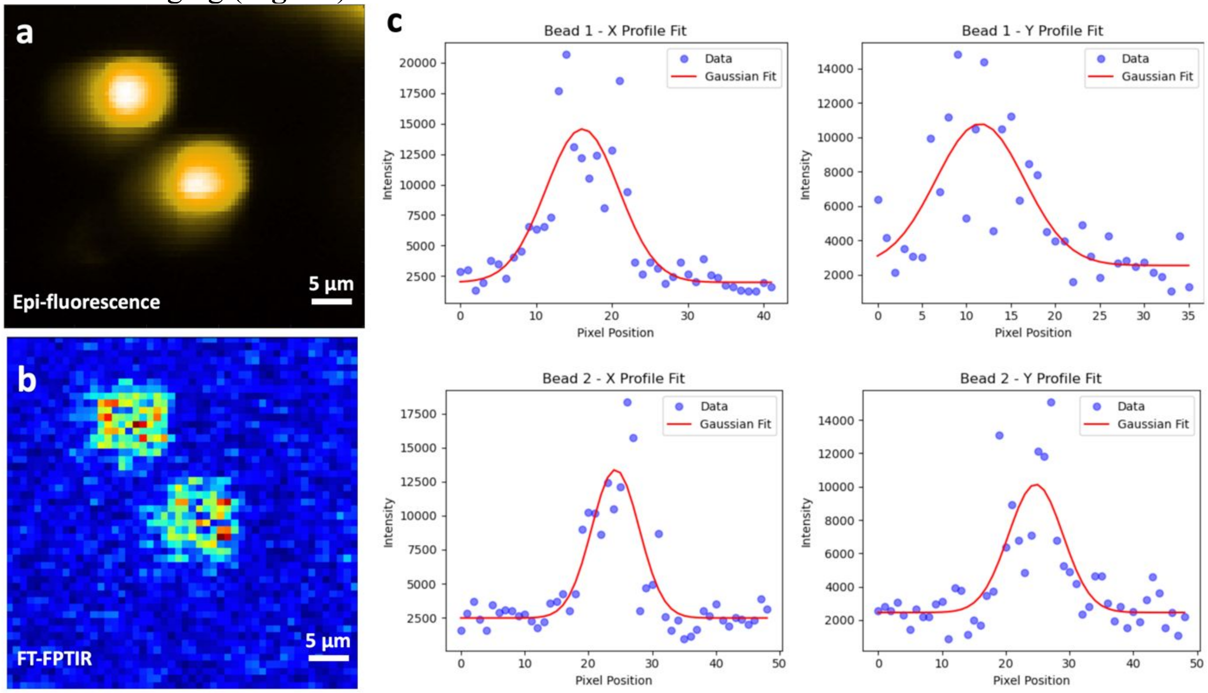

Figure S8. Epi-fluorescence (a) and FT-FPTIR (b) imaging of 10 µm polystyrene beads and bead profile estimate (c).

An example of an FT-FPTIR interferogram

A sample interferogram for the fluorescence-detected modality collected using the home-built instrument is provided for additional reference.

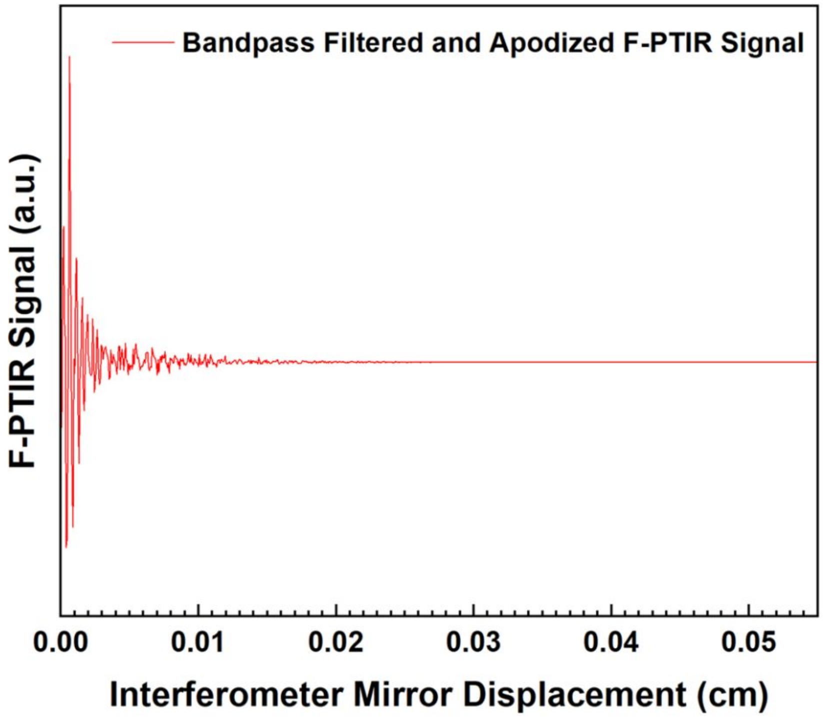

Figure S9. A synchrotron F-PTIR interferogram of the silica gel sample.

Bright-field image for Figure 3.

The following bright-field image corresponds to the FoV shown in **Figure 3** of the main manuscript with the red box corresponding to the FoV used for FTIR and FT-PTIR imaging:

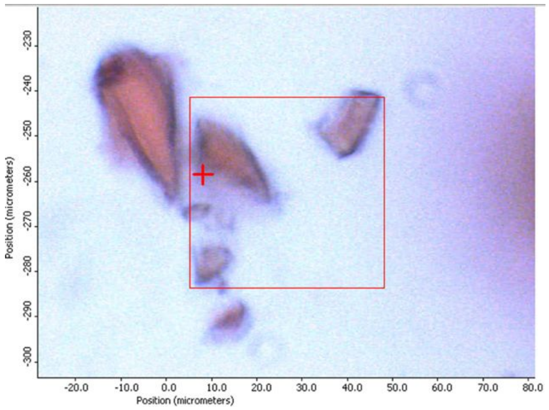

Figure S10. Bright field image of rhodamine-labeled silica gel particles, indicating the field-of-view (red square) used for FTIR and FT-PTIR imaging shown in the main manuscript.

4. Additional supporting data for mid-IR spectroscopic imaging of mouse brain tissue sections.

A single full-range synchrotron FTIR spectrum of the brain tissue brain sample is provided in **Fig. S11** with the most relevant biological peaks labeled. The corresponding peak origins are listed in **Table S2**.

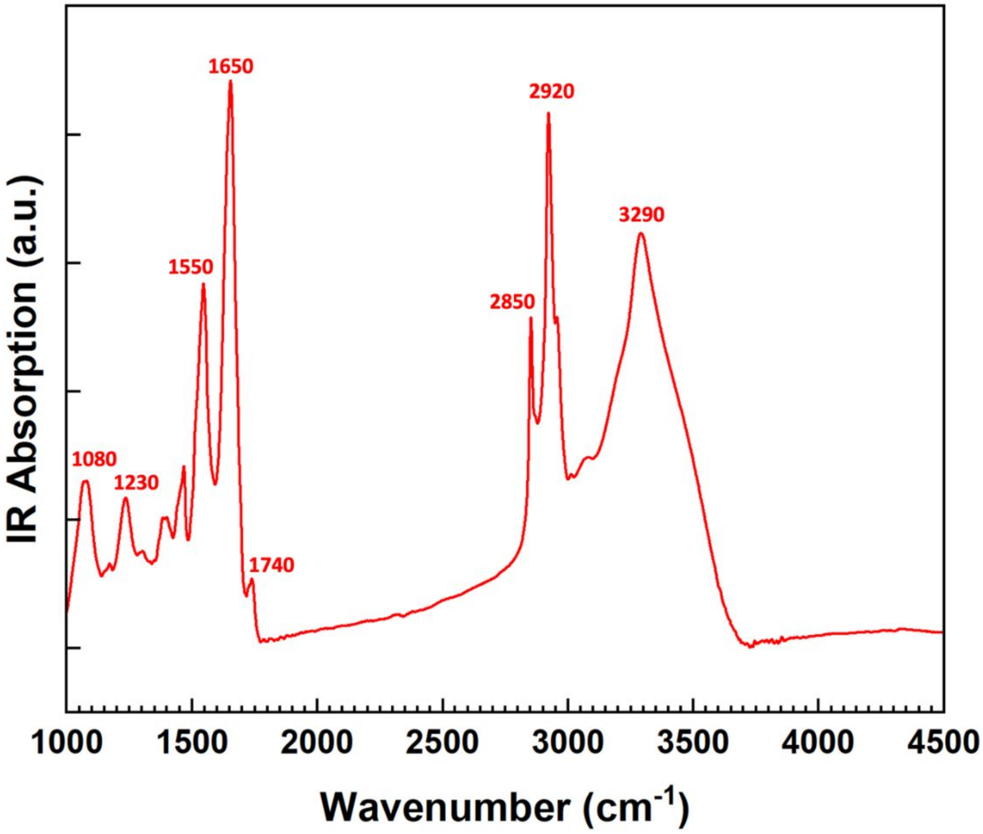

Figure S11. A sample synchrotron FTIR spectrum of the striatum of a mouse brain tissue section. The striatum represents a lipid-rich region of the brain, as evidenced by the relative high intensity of the CH stretch region.

Table S2. Relevant FTIR absorption peaks of biomolecules

Peak Position (cm^{-1})	Corresponding Vibration and Biomolecule Class
≈ 1080	Carbohydrates (CO-O-C bond)
≈ 1230	Nucleic Acids (phosphate group)
≈ 1550	Proteins (Amide II, N-H)
≈ 1650	Proteins (Amide I, C=O)
≈ 1740	Lipids (C=O)
≈ 2850	Lipids (CH_2 stretch)
≈ 2920	Lipids (CH_2 stretch)
≈ 3300	Proteins (Amide A, N-H), also OH groups

Additional single-frequency absorption maps for the brain tissue FoV shown in **Fig. 4** in the main manuscript for the abovementioned absorption peaks are provided in **Fig. S12** below. The maps were normalized by the closest protein absorption peak to normalize for etalon effects, film thickness, and wavelength changes in order to get potential insights into the spatial distribution of proteins and lipids within the tissue sample.

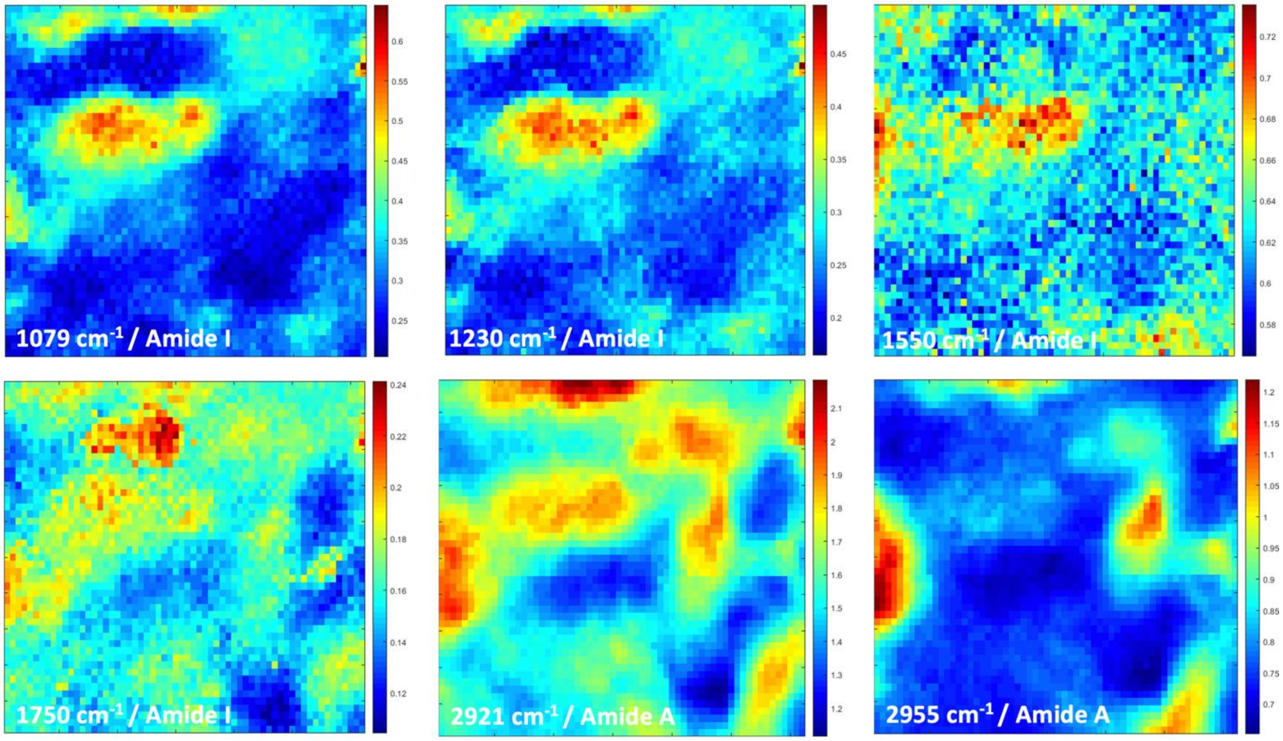

Figure S12. Single-frequency synchrotron FTIR absorption maps for the main spectral signatures of biomolecules.

While no clear contrast between the cells and the extracellular tissue could be observed for spectral bands associated with nucleic acids and carbohydrates, FT-PTIR-guided cell-specific analysis revealed the potential decrease in lipids to protein in the regions identified as cell bodies from the fluorescence imaging, as discussed in the main manuscript.

Following this observation, the FTIR signal originating from the protein peaks was normalized by the adjacent lipids to reveal potential spatial heterogeneity of the lipid distribution. As shown in **Fig. S1e**, the concentration of lipids seems to be lower within cell bodies relative to the extracellular tissue. The difference is most obvious when normalizing the lipid CH-stretch with the Amide A band with this spectral map. The corresponding image resembles the immunofluorescence image of cell nuclei shown in the main manuscript, indicating the possibility of fully digital labeling of cell bodies. An increased concentration of lipids in the extracellular region is additionally confirmed by calculating the ratio of intensities of the lipid peaks to adjacent protein peaks (**Table S2**).

Table S3. Relative ratios of lipids to proteins absorption bands in the fingerprint region and in the CH-stretch region of the IR spectrum.

Cell Label	1750/1655 cm^{-1} Ratio	2920/3290 cm^{-1} Ratio
**A**	0.11	1.30
**B**	0.11	1.30
**C**	0.12	1.31
**D**	0.13	1.31
**E**	0.14	1.60
**F**	0.12	1.33
**Extracellular Tissue**	0.16	1.76

Furthermore, additional ratiometric analysis of the lipid spectral peaks revealed spatial heterogeneity for the ratio of lipid -CH_2 to -CH_3 groups, which could potentially indicate the difference in carbon chain length for lipids accumulating in different parts of the striatum.

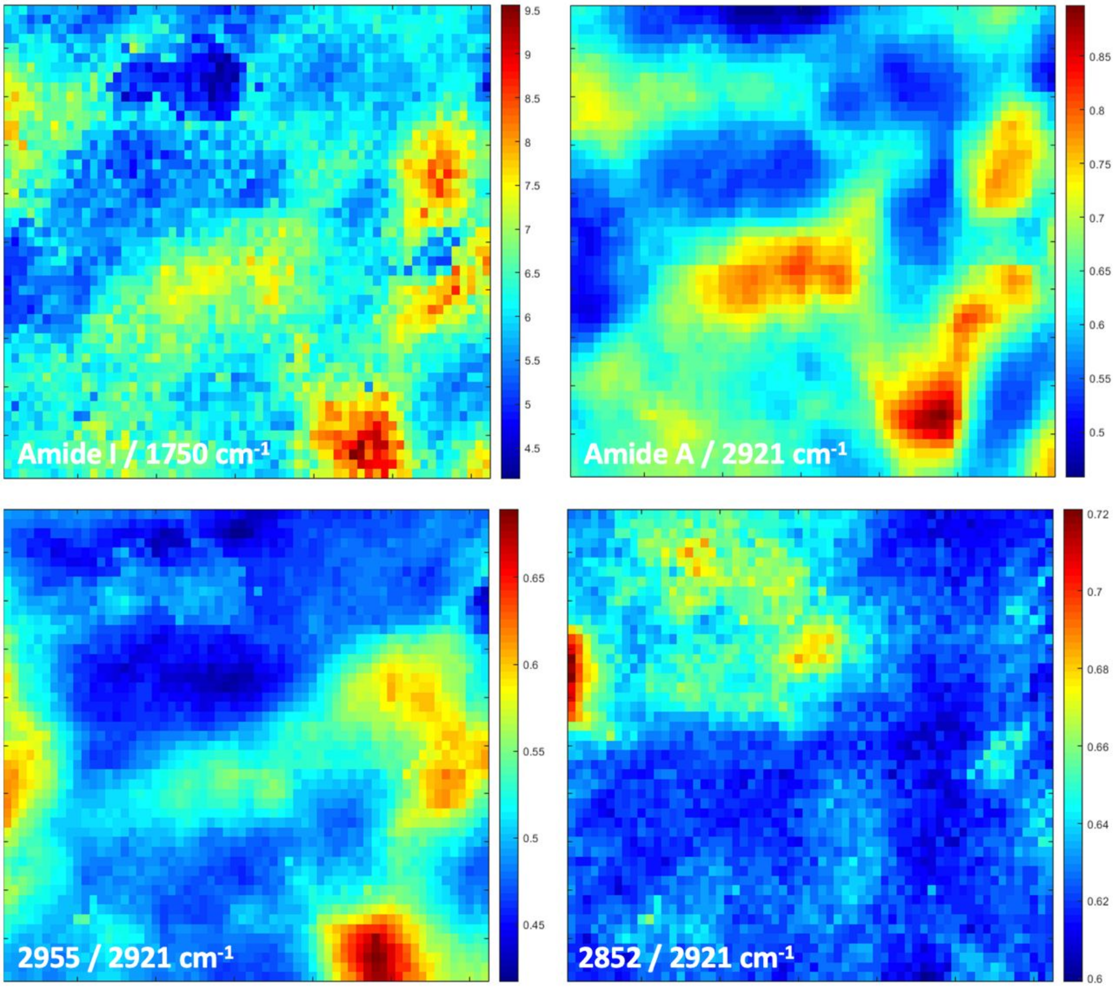

Figure S13. Ratiometric analysis of synchrotron FTIR absorption maps of the brain tissue sample. **Top row** – Ratio maps of proteins to lipids absorption in the fingerprint and CH stretch regions. **Bottom row** – Ratio maps for different signatures absorption peaks of lipids.

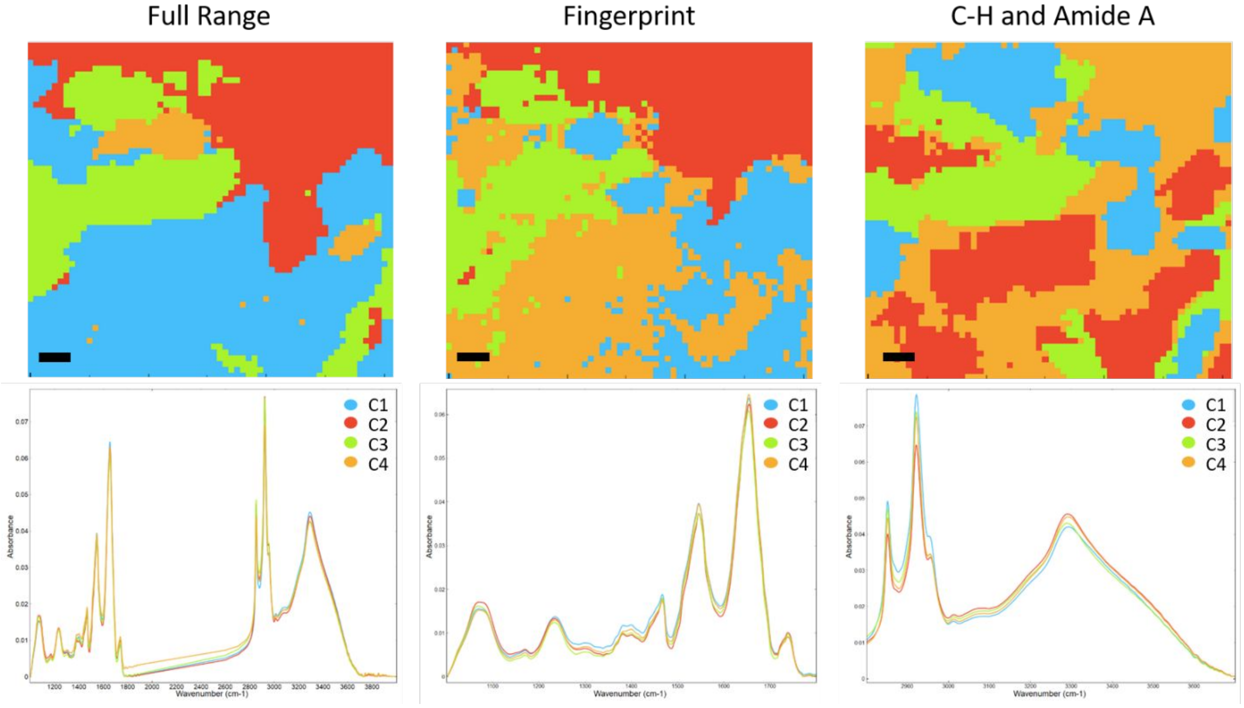

Figure S14. *K*-means clustering analysis of synchrotron FTIR absorption maps of the brain tissue sample. **Top row** – *K*-means clustering based on the full spectral range, the fingerprint region only (1000-1800 cm^{-1}), and the region covering the C-H and Amide A bands (2800-3700 cm^{-1}). Scale bars are 5µm. Prior to *K*-means analysis, the spectra were vector-normalized across the entire spectral region. **Bottom row** – Average spectra of the clusters color coded according to the colors in the top row. The region covering the C-H and Amide A region clusters similarly to the distribution of the Amide A/ 2921 cm^{-1} ratio in Figure S13 above. The clustering indicates regions of lower lipid content and correlates with locations of the fluorescently-labeled nuclei, but there is not a one-to-one correspondence between the two.
